# Kinetic MUNANA assay reveals functionally relevant antibody epitopes on Influenza A virus neuraminidase

**DOI:** 10.1038/s44298-025-00123-y

**Published:** 2025-05-10

**Authors:** Ilya V. Smirnov, Danica F. Besavilla, Karin Schön, Hannes Axelsson, Davide Angeletti

**Affiliations:** 1 Department of Microbiology and Immunology, Institute of Biomedicine, University of Gothenburg, Gothenburg, Sweden; 2 SciLifeLab, Institute of Biomedicine, University of Gothenburg, Gothenburg, Sweden

**Keywords:** Antibody generation, Antibody isolation and purification, Influenza virus

## Abstract

Influenza A virus neuraminidase (NA) is drawing attention as a target for vaccine development. In this study, we propose kinetic MUNANA assay as a tool to identify monoclonal antibodies (mAbs) that specifically target functional epitopes on NA. By analyzing changes in the parameters of the Michaelis-Menten curve (Km and Vmax), we revealed distinct mechanisms of Ab-mediated inhibition. Additionally, we developed a web-based application facilitating efficient processing of the assay results and enabling statistical inference. We employed the kinetic MUNANA assay to test newly developed mAbs targeting NA of the widely used PR8 H1N1 strain. Among these, mAbs with strong effect on NA enzymatic parameters were more likely to select for escape mutants that had a substantial impact on the overall enzymatic activity of the virus. In summary, when combined with ELLA, kinetic MUNANA is a rapid method to profile the putative binding site and the effect of NA-specific mAbs.

## Introduction

Influenza A virus (IAV) poses a persistent public health threat due to its constant antigenic drift, leading to the annual emergence of new variants^1^. Mass vaccination remains the most effective strategy to mitigate the morbidity and mortality associated with influenza outbreaks. Current vaccines target the major viral surface glycoprotein, hemagglutinin (HA), however they show variable efficacy, hence the need to explore alternative targets. In this context, Neuraminidase (NA), the second most abundant surface glycoprotein of IAV, has gained prominence^2^, as low-molecular-weight NA-inhibiting (NI) drugs (such as peramivir, zanamivir, oseltamivir, etc.) have demonstrated high efficacy in controlling active viral infections and alleviating symptoms^3,4^.

Anti-HA and anti-NA antibodies (Abs) provide protection through distinct mechanisms, reflecting the differing roles of HA and NA in viral replication^5^. HA facilitates viral entry into host cells by binding to terminal sialic acids on cell surface glycans and plays a crucial role in endosomal membrane fusion, leading to viral uncoating^6^. As a result, HA-specific Abs neutralize viral particles and block infection of host cells. In contrast, NA is important for virion motility and enables the release of progeny virions by cleaving sialic acids from glycans on the surface of infected cells^7–10^. Consequently, NA-specific Abs mostly limit viral spread and reduce disease severity, but are also capable of inhibiting initial infections^11–15^. Importantly, NA is significantly less abundant but relatively more conserved than HA^16,17^, suggesting that NA-targeting Abs may provide more durable protection against diverse viral strains.

The protective efficacy of NA-specific Abs often correlates with their ability to inhibit NA enzymatic activity, still they can also mediate Fc-dependent effector functions^18–21^. NI monoclonal antibodies (mAbs) have demonstrated effectiveness in protecting mice from lethal influenza infections^22^. Furthermore, serum levels of NI Abs are independently associated with protection against IAV infection in humans^14,22,23^. Nevertheless, the role of distinct anti-NA Abs, targeting different epitopes, in host protection is less studied. Indeed, while antigenic structure of HA is very well defined, the antigenic sites of NA are poorly described, with extensive studies done only for one N1 and one N2^8,24^. Defining epitopes targeted by Abs would greatly facilitate future work. Furthermore, mAbs with defined epitope footprints, specific for Influenza A/Puerto Rico/8/1934 H1N1 (PR8), the most widely used laboratory IAV strain, would benefit the research community.

NA-inhibitory properties of antiviral drugs and Abs are evaluated using laboratory assays such as MUNANA assay, NA-Star assay, and enzyme-linked lectin assay (ELLA). The MUNANA and NA-Star assays use small fluorescent and chemiluminescent substrates, respectively, making them particularly suited for assessing antiviral drugs, as they efficiently compete. However, these methods are less sensitive than ELLA in detecting NI mAbs, as small substrate molecules can still access the enzyme’s active site even in the presence of most bound Abs, with the notable exception of rare broadly neutralizing Abs specific for the NA active site^19^. In contrast, ELLA uses heavily sialylated fetuin (~40 kDa) as a substrate^25^, making it better for evaluating Ab-mediated inhibition. The MUNANA assay can be performed in two formats: an endpoint assay or a kinetic assay. The endpoint assay measures IC_50_ values, with lower values indicating stronger inhibition. mAbs that have demonstrated inhibitory effects have a high likelihood of mitigating the diseases symptoms in in vivo experiments^26^. On the other hand, the end-point enzymatic assay does not provide any information regarding the mechanism of Ab-mediated inhibition of NA function. The kinetic assay provides deeper mechanistic insights by estimating the Michaelis-Menten parameters, Km and Vmax^27^. Changes in these parameters in the presence of an inhibitor can reveal inhibition´s mechanism, offering a detailed understanding of how NA enzymatic activity is modulated.

Here, we hypothesized that the kinetic MUNANA assay, used alongside traditional ELLA, could effectively characterize how mAbs influence NA enzymatic function. To test this, we developed a panel of mAbs targeting the NA of the IAV laboratory strain PR8, analyzed their effect by ELLA and kinetic MUNANA assay and mapped their epitopes by classical escape selection. In addition, to streamline the analysis of kinetic MUNANA data, we created an openly available web-based Shiny application to calculate Km and Vmax values and provide statistical insights into the effects of mAbs on these parameters.

## Results

### Anti-NA mAbs bind to distinct antigenic sites

To facilitate the investigation of NA activity and functional antigenic sites, we set out to generate and characterize a panel of mAbs targeting the widely used PR8 virus. To produce anti-NA mAbs, we first infected BALB/c mice with PR8 virus and subsequently boosted them i.p. with soluble, recombinant NA (rNA). rNA exhibited enzymatic activity (Supplementary Fig. 1A), owing to the stabilization of its tetrameric structure through the incorporation of a tetrabrachion, self-tetramerizing, motif^28,29^. Given the relatively low abundance of NA on viral particles compared to the highly immunogenic HA, viral particles could not be used for screening of serum and hybridomas. Hence, to test NA-specific response, we used two ELISA assays: 1) an indirect ELISA for rNA and 2) a cell ELISA based on fixed murine NCTC929 cells stably expressing full-length membrane-associated NA fused with EGFP. These EGFP-NA-expressing cells retained sialidase activity (Supplementary Fig. 1B). By using these assays, we could confirm that our immunization regime effectively induced robust NA-specific antibody responses in the animals (Supplementary Fig. 1C).

**Fig. 1:**
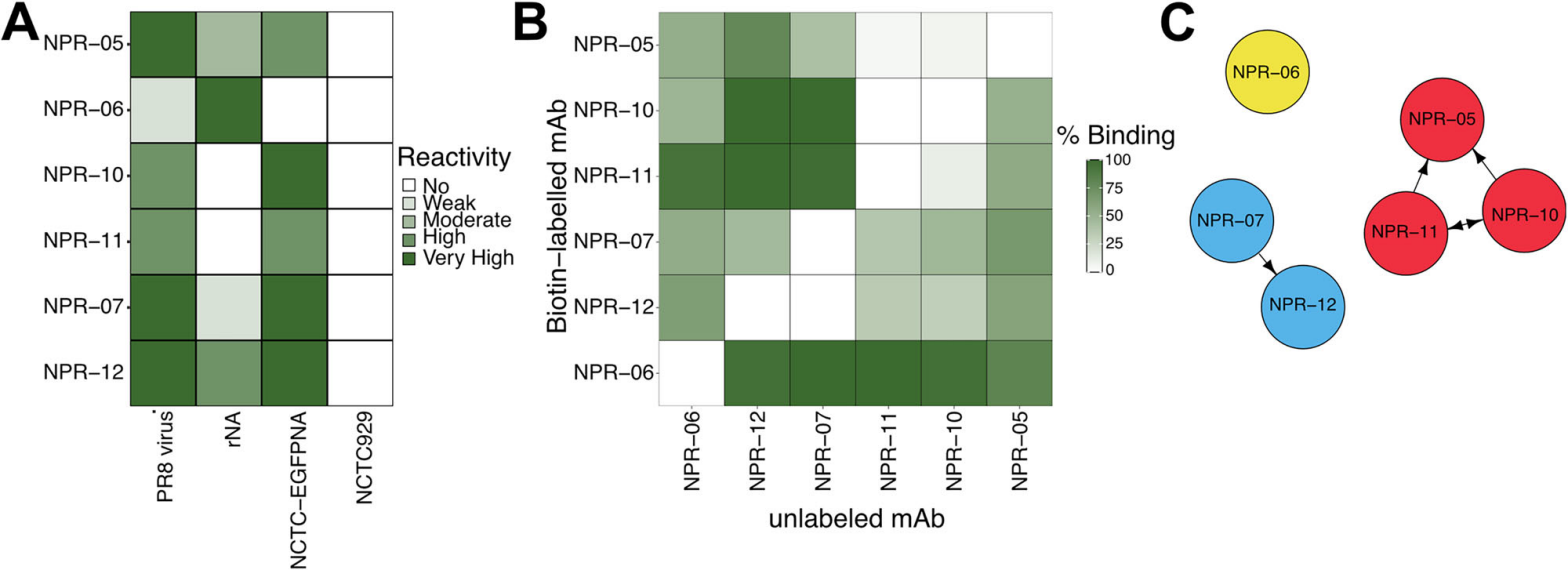
Characteristics of anti-NA mAbs. **A** Immunoreactivity of IgG2a mAbs (10 μg/ml) in ELISA with NA from various sources: adsorbed PR8 virus particles, recombinant protein, or expressed on the membranes of NCTC929 cells as an EGFP fusion protein. Reactivity with intact cells is shown as a specificity control. A relative scale is used, as the assays have different sensitivities (Supplementary Table 2). **B**, **C** Competitive ELISA results. Binding of biotin-labeled mAb to PR8 virus particles in the presence of unlabeled counterparts (10 μg/ml). OD data was log-transformed before calculations. **B** Mutual arrangement of epitopes recognized by the mAbs defined by using community detection based on edge betweenness (Newman-Girvan) with a threshold of >70% inhibition (*N* = 3). **C** Connected mAbs belong to the same community and arrow indicates inhibition direction.

Spleen were then fused with Sp2/o myeloma cells to generate hybridomas. Hybridomas were further cultivated if they produced mAbs reacting either to rNA or EGFP-NA on cells and having no binding with intact NCTC929 cells. Finally, we developed a panel of anti-NA mAbs consisting of 13 members (named as **N**A **PR**8 - NPR followed by a number). Six hybridoma cultures (NPR-02, -03, -04, -09, -14 and -20) produced IgM mAbs, one an IgA mAb (NPR-15), while six others (NPR-05, -06, -07, -10, -11 and 12) produced IgG2a mAbs. To assess the immunological reactivity of the mAbs, their binding was tested in ELISA using viral particles, rNA, and fixed NA-expressing cells as antigens. All IgM and the IgA were weakly reactive in ELISA with adsorbed viral particles despite showing good reactivity with NA molecules in cell ELISA (Supplementary Fig. 2). mAb NPR-06 showed high reactivity to rNA but weak reactivity with viral particles and no reactivity in cell ELISA (Fig. 1A, Supplementary Fig. 3 and Table 1). The other five IgG2a mAbs in our panel (NPR-05, -07, -10, -11, and -12) showed high reactivity with membrane bound-NA and effectively recognized their epitopes on viral particles. NPR-10 and NPR-11 did not bind rNA possibly due to conformational changes in their epitopes caused by the adsorption of the antigen onto the solid phase. For subsequent experiments, we exclusively used IgG2a mAbs, as they demonstrated the strongest reactivity with viral particles.

**Table 1:** Key properties of IgG2a anti-NA mAbs established in the study

mAbs	Apparent Kd (nM)^a^	IC50 (nM)^b^	Vmax (μM/min)^c^	Km (μM)^c^	Type of inhibitor^c^
NPR-05	0.01	0.176	0.7918	36.37	Mixed-type
NPR-06	104.27	0.976	0.2817	13.68	Steric
NPR-07	2.81	0.393	0.2951	34.39	Competitive
NPR-10	11.09	0.436	0.2995	23.85	Steric
NPR-11	32.11	4.040	0.2346	21.95	Non-competitive
NPR-12	0.85	1.900	0.2989	15.36	Steric

^a^Determined by ELISA.

^b^Determined by ELLA.

^c^Determined by kinetic MUNANA. Control mAb Vmax = 0.2832 μM/min, Km = 18.11 μM.

To map the spatial relationships between the epitopes targeted by the mAbs, we performed a competitive ELISA using PR8 virus particles as the antigen and calculated the % binding of each biotin-labelled mAb in the presence of unlabelled competitor mAb (Fig. 1B). The results indicated that the mAbs bind to three antigenic sites on the NA surface, defined by NPR-07/12, NPR-05/10/11 and NPR-06, with some mAbs exhibiting mutual inhibition. Within each antigenic site, mAbs showed strong mutual inhibition, suggesting that their binding sites are closely related or overlapping. NPR-05 exhibited partial inhibition of mAbs in the NPR-10/11 subcluster, indicating that its epitope is located near, but not completely overlapping to the antigenic site targeted by NPR-10 and NPR-11 (Fig. 1C). Finally, consistent with its unique binding properties (Fig. 1A), NPR-06 bound to a completely distinct antigenic site (Fig. 1C).

In summary, we report the development of several hybridoma clones against the widely used laboratory adapted PR8 strain which bind to distinct antigenic sites.

### Diverse effects of anti-NA mAbs on NA enzymatic activity detected by kinetic MUNANA assay

**Fig. 2:**
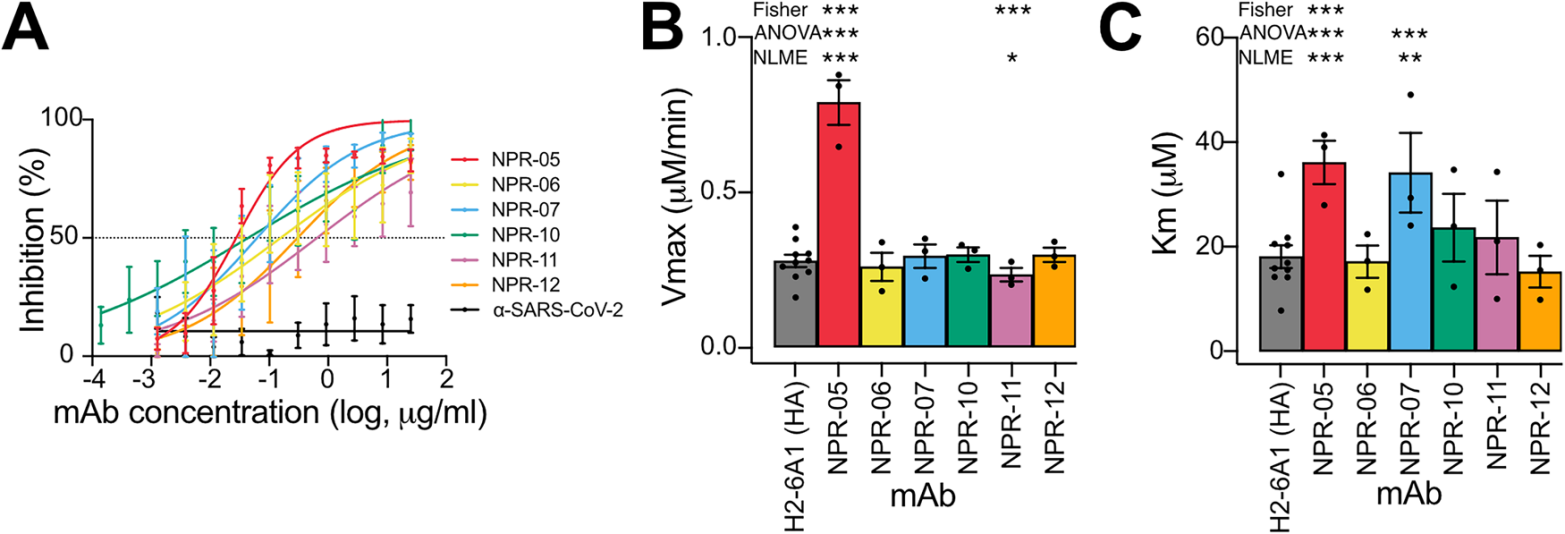
NA-inhibitory properties of mAbs. **A** Comparison of mAb (10 μg/ml) inhibition potency in the ELLA assay. Mean values are shown and error bars denotes SEM, (*N* = 3). Effects of mAbs on NA enzymatic activity assessed in kinetic MUNANA assay. Shown are estimates of Vmax (**B**) and Km (**C**). Data are shown as mean ± SEM, (*N* = 3). Common color scheme was used in all panels.

To verify the functionality of the mAbs, we tested their NI capacity by endpoint ELLA assay. All mAbs, including NPR-06, demonstrated NI with varying IC_50_ capacity with NPR-05, NPR-07 and NPR-10 having the highest NI potency (Fig. 2A, Table 1). However, it is well know that NI can be mediated via several distinct mechanisms^30,31^. Therefore, we reasoned that by using kinetic MUNANA assay we would be able to more precisely infer the binding area of the mAbs. We adapted and further corrected the approach suggested by Marathe^27^ and developed an R-based, Shiny app, which we made freely available online, to easily analyze data and allow for statistical comparison of several samples.

As hypothesized, opposite to ELLA, NA enzymatic activity in the kinetic MUNANA assay was differentially influenced by distinct NI mAbs (Fig. 2B, C and Supplementary Table 1). NPR-05 enhanced the enzymatic activity of viral NA, increasing both Vmax and Km, consistent with it being a mixed-type inhibitor of the enzyme. NPR-07 increased Km without affecting Vmax, indicative of competitive inhibition. In contrast, NPR-11 reduced the overall catalytic activity of NA, as evidenced by a decrease in Vmax, while Km remained unchanged, aligning with a non-competitive inhibition. Finally, NPR-06, NPR-10 and NPR-12 did not significantly affect the Michaelis-Menten parameters, suggesting that their inhibitory activity detected by ELLA resulted from steric hindrance (Table 1). Interestingly, the effects of mAbs on NA enzymatic properties, as identified by the kinetic MUNANA assay, did not fully correlate with the competitive ELISA results. This discrepancy suggests that the competitive ELISA lacks sufficient resolution, limiting its ability to distinguish between mAbs with different functional properties that target closely located epitopes.

### NPR-05 and NPR-07 bind to distinct epitopes, close to NA active site

To verify the binding sites of the mAbs and correlate these with kinetic MUNANA results, we mapped mAb epitopes by selecting viral escape mutants. We performed six consecutive rounds of virus selection to generate escape mutants, starting at 0.25 times ELLA IC_50_ (Table 1) and doubling mAb concentrations with each round.

This approach successfully yielded three NA mutants for NPR-05 (N185D, D233N, K206E), three for NPR-07 (T317K, D326G and D326G/V378I) and two for NPR-12 (S259Y and Y324H). Most of the mutant viruses also carried mutations in the HA segment (Table 2). However, no NA mutations were identified after selection with NPR-06, NPR-10 and NPR-11; indeed, NPR-06 and NPR-10 yielded only HA mutations, which may have either compensated for NA inhibition by mAbs^32,33^ or arisen due to other selective pressure, not controlled in the experiment. NPR-11 did not generate any detectable mutation in either HA or NA genes (Table 2). Notably, NPR-05 and NPR-07 exhibited the strongest impact on NA enzymatic activity in both ELLA and the kinetic MUNANA assay (Fig. 2). Importantly, these two mAbs were clearly distinguished from the others by their ability to increase Km values in the kinetic MUNANA assay (Table 1). Furthermore, NPR-05 and NPR-07 also were among the strongest binders according to apparent binding affinities (Table 1); yet NPR-12, the second strongest binder, did not lead to the selection of true escape mutants.

**Table 2:** NA and HA mutations (H1 numbering) after six passages in the presence of indicated anti-NA mAbs

mAb	Clone	NA mutations	HA mutations
NPR-05	1	D233N	
	2	N185D	N261D
	3	N185D	H143P
	4	K206E	
NPR-06	1		F258S
NPR-07	1	T317K	T397S
	2	T317K	N303D
	3	D326G	
	4	D326G/V378I	
NPR-10	1		N261D
	2		N210T
	3		K187E
NPR-12	1	S259Y	
	2	Y324H	

To confirm that identified mutations to be within the antibody footprints and to eliminate potential confounding effects from HA mutations, we rescued all NA mutant viruses on a PR8 WT backbone and evaluated the impact of NA mutations on mAb-mediated effects.

**Fig. 3:**
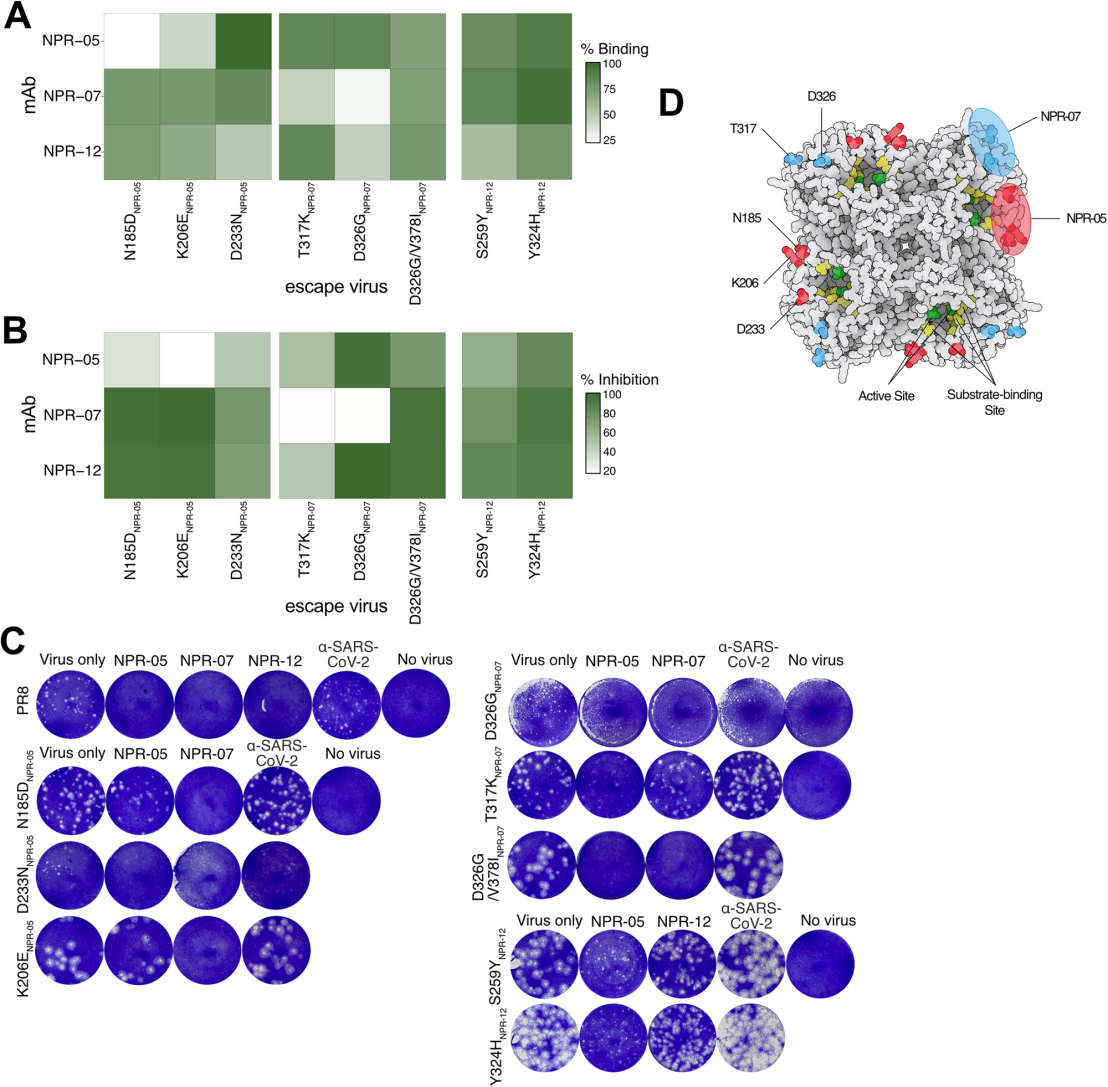
NA escape mutations following passaging of PR8 virus in the presence of mAbs. **A** Mean AUC reactivity of escape mutants with mutated viruses in ELISA, (*N* = 3). The mAbs used to select the mutants are indicated at the top of each panel, while the mAbs tested in ELISA are listed on the left. NA-mAb NA-1C1, was used to normalize virus amount on the plate and binding of each mAb to WT PR8 virus was set to 100%. **B** Mean of NA-inhibitory properties of the escape mutants in the presence of mAbs, (*N* = 3). mAbs were normalized to an irrelevant IgG mAb, anti-SARS-CoV-2 mAb#27, which was set to 0% inhibition. **C** Representative experiment of plaque reduction assays. Plaques formed by the escape mutants in the presence of mAbs, an irrelevant IgG mAb, anti-SARS-CoV-2 mAb#27, was used as a control (*N* = 3). **D** Location of amino acid replacements in true escape mutants mapped onto the homotetrameric NA (PDB: 6D96). Approximate binding sites of NPR-05 and NPR-07 mAbs are shown relative to the active site and substrate-binding site.

Firstly, NPR-05 and NPR-07 exhibited reduced binding to their respective escape mutants in ELISA (Fig. 3A), except for the D326G/V378I double mutant, confirming that these amino acid substitutions contribute to their epitopes. In contrast, NPR-12 retained its binding to the mutants selected in its presence, suggesting that these mutations did not disrupt its epitope but instead facilitated escape through alternative mechanisms.

Secondly, the NI activity of NPR-05 and NPR-07 was also reduced in ELLA assay when tested against their corresponding escape mutants (Fig. 3B), further emphasizing the critical role of these residues in mAb binding. Meanwhile, NPR-12 maintained its NI activity against its selected mutants, consistent with the ELISA findings.

Lastly, we performed a plaque reduction assay to assess whether mAbs could block viral infection of the respective escape mutants. As expected, NPR-05, NPR-07, and NPR-12 effectively blocked plaque formation upon PR8 WT infection. However, most escape mutants formed plaques despite treatment with their corresponding mAbs, while plaques were absent in wells treated with unrelated mAbs (Fig. 3C and Supplementary Fig. 4). Interestingly, Y324H_NPR-12_ and S259Y_NPR-12_ viruses continued to grow in the presence of both NPR-12 and even NPR-05, contradicting the ELISA and ELLA results. Additionally, D233N_NPR-05_ and D326G_NPR-07_ displayed smaller plaque sizes, suggesting potential growth defects, making it more challenging to confirm escape.

Consequently, five amino acid replacements were mapped to binding sites of two mAbs (NPR-05: N185D, D233N, and K206E; NPR-07: T317K and D326G). Highlighting the locations of these amino acids on the NA structure revealed that these mAbs bound to distinct epitopes localized near the enzyme’s active site (Fig. 3D), confirming the ELISA competition results (Fig. 1B, C) and partially explaining the kinetic MUNANA results (Fig. 2B, C).

### NPR-05 and NPR-07 target functionally relevant NA epitopes

**Fig. 4:**
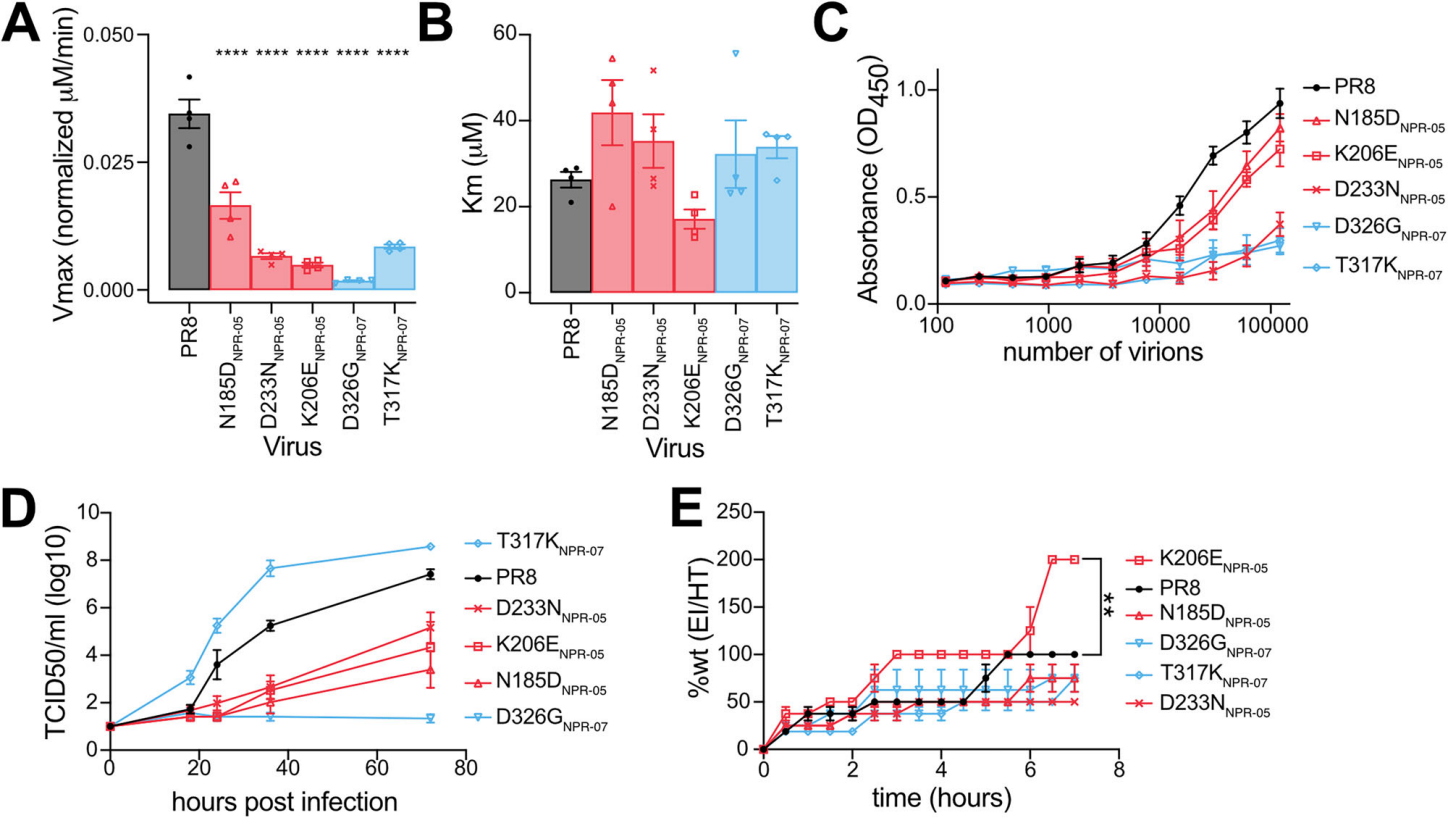
Functional relevance of NA epitopes affected by escape mutations. **A**, **B** Enzymatic properties of NA in escape mutants assessed using the kinetic MUNANA assay: Vmax (**A**) and Km (**B**) values. Vmax was normalized according to number of virions per sample. Data are shown as mean ± SEM, (*N* = 4). Significant differences were determined with One-way ANOVA with multiple comparisons and Dunnett testing. **** denotes a *p*-value of ≤0.0001. **C** NA activity of witld-type and escape mutants assessed through ELLA. It was performed using normalized number of virions, starting at 120,000 then serially diluted two-fold per virus. Mean values are shown and error bars denotes standard deviation (*N* = 6). **D** Growth curves of wild-type and escape mutant viruses in MDCK cells. Significant differences were determined with Two-way ANOVA with multiple comparisons and Dunnett testing. Differences in viral replication was seen between the mutant virus compared to PR8 WT virus at the 18 h (* T317K_NPR-07_), 24 h (*** N185D_NPR-05_, K206E_NPR-05_, D326G_NPR-07_; ** D233N_NPR-05_, T317K_NPR-07_), 36 h (**** N185D_NPR-05_, D233N_NPR-05_, K206E_NPR-0_, D326G_NPR-07_, T317K_NPR-07_), and 72 h timepoint (**** N185D_NPR-05_, D233N_NPR-05_, K206E_NPR-0_, D326G_NPR-07_). * denotes a *p*-value of ≤0.05, ** denotes a *p*-value of ≤0.01, *** denotes a *p*-value of ≤0.001, **** denotes a *p*-value of ≤0.0001. Geometric means are shown, and error bars denotes standard error of the mean, (*N* = 12). **E** Elution properties of viral escape mutants normalized to the same Vmax value, compared to wild-type PR8 virus in an RBC virus elution assay. Dots are mean ± SEM. Significant differences were determined with One-way ANOVA with multiple comparisons and Dunnett testing on the areas under the curve of the data. ** denotes a *p*-value of ≤0.01. (*N* = 4).

To evaluate the functional significance of the epitopes targeted by NPR-05 and NPR-07 mAbs, we analyzed the enzymatic properties of the rescued mutants, normalizing the viral particle counts based on hemagglutination activity, since all viruses carried the same WT HA segment. Both ELLA and kinetic MUNANA assay detected a reduction in enzymatic activity in the mutant viruses compared to WT PR8 (Fig. 4A, B, Table 3). However, the kinetic MUNANA assay revealed that this decrease resulted from a reduction in overall enzymatic efficiency (Vmax) rather than a decrease in substrate affinity, as indicated by an unchanged Km value. These findings suggest that the mutants exhibit structural alterations near the NA active site (Fig. 3D), which restrict substrate turnover while preserving substrate affinity.

**Table 3:** Summary of selected NA mutations and their properties

NA	ELLA activity (% of wt)^a^	Vmax (nM/min)^b^	Km (uM)	Change in MDCK growth^c^	Change in RBC release^c^
wt	100	34.5	26.3	–	–
N185D_NPR-05_	80.56	16.5	41.9	↓	↓
K206E_NPR-05_	72.49	4.88	17.1	↓	↓
D233N_NPR-05_	28.09	6.62	35.3	↓	↑
T317K_NPR-07_	29.79	8.47	33.9	↑	↓
D326G_NPR-07_	31.48	1.75	32.2	↓	↓

^a^Change of activity in ELLA compared to wild-type PR8 virus (60,000 virions) set as 100%.

^b^Values of overall catalytic power with 60,000 virions used.

^c^Change compared to wild-type PR8 virus. ↓ indicates decreased growth/release while ↑ indicates increased growth/release.

To evaluate how these altered enzymatic properties influenced viral fitness, we measured the growth dynamics of mutant viruses in MDCK cells (Fig. 4D, Table 3). Most mutants displayed lower growth than wild-type PR8, except for T317K_NPR-07_, which exhibited faster initial replication, but plateaued at the same level as wild-type PR8. Additionally, most escape mutants showed reduced virion release from sialic acid, compared to wild-type PR8 (Fig. 4E, Table 3). Surprisingly, K206E_NPR-05_ reached complete elution after 3 h despite the poor enzymatic measured in MUNANA (Fig. 4E). However, it should be noted that this mutant maintained one of the highest ELLA activities, after PR8 WT (Fig. 4A)

Collectively, our findings indicate that NPR-05 and NPR-07 mAbs target functionally important epitopes, as amino acid replacements in their binding site impaired or altered NA function.

## Discussion

In this study, we present an approach for the functional characterization of anti-NA mAbs, combining ELLA with kinetic MUNANA assay. This integrated strategy enables the initial categorization of mAbs into three main groups: non-inhibitors, steric inhibitors, and functional inhibitors, without requiring prior knowledge of their epitope specificity. Here, we defined non-inhibitors as mAbs that do not alter NA activity in either assay. Steric inhibitors interfere with NA’s interaction with high-molecular-weight substrates, resulting in reduced enzymatic activity in ELLA, while leaving the kinetic parameters measured in the MUNANA assay unaffected (NPR-06, NPR-10, and NPR-12). In contrast, functional inhibitors induce measurable changes in the enzymatic properties of the NA active site, as revealed by altered kinetic parameters in the MUNANA assay using a low-molecular-weight substrate (NPR-05, NPR-07, and NPR-11).

The kinetic MUNANA assay not only identifies functional inhibitors but also enables their further classification based on their mode of action. Competitive inhibitors selectively increase Km (NPR-07), indicating reduced MUNANA substrate binding affinity, while non-competitive inhibitors decrease Vmax (NPR-11), reflecting impaired overall catalytic efficiency. Uncompetitive inhibitors reduce both apparent Km and Vmax, whereas mixed-type inhibitors (NPR-05) increase Km while simultaneously decreasing Vmax^34–36^. Mechanistically, both steric inhibitors and functional inhibitors that reduce Km are expected to interfere with substrate binding to NA. However, the binding sites of competitive inhibitors are expected to be located closer to the NA active site. Due to its smaller molecular size compared to fetuin used in ELLA, the MUNANA substrate provides enhanced resolution for detecting mAb binding in the vicinity of the active site. One key finding is that only the combination of ELLA and kinetic MUNANA assay can provide a comprehensive understanding of mAb-mediated inhibition of NA activity. ELLA detects mAbs that inhibit NA either by disrupting active site function or by blocking interaction with large sialylated proteins, but it does not distinguish between these mechanisms. In contrast, the kinetic MUNANA assay specifically identifies mAbs that interfere with active site function but cannot detect those that inhibit NA binding to high-molecular-weight substrates without affecting enzymatic activity.

As part of our study, we isolated 13 new hybridoma clones producing PR8 NA-specific mAbs, which can serve as valuable tools for future studies on this virus. To the best of our knowledge, a panel of mAbs against PR8 NA has not been described, although some anti-NA mAbs cross-reacting with the PR8 strain are known^37,38^. We focused on six IgG2a mAbs, which exhibited strong reactivity with viral particles, for further analysis. All IgG2a mAbs exhibited varying degrees of NI activity in ELLA. However, three of these mAbs (NPR-06, NPR-10, and NPR-12) did not alter active site functionality in the kinetic MUNANA assay. Three other IgG2a mAbs (NPR-05, NPR-07, and NPR-11) demonstrated the ability to modify the enzymatic activity of the NA active site.

The kinetic MUNANA assay provided a possible mechanistic explanation for mAb-mediated inhibition of NA enzymatic activity. The addition of NPR-07 increased Km without affecting Vmax, suggesting that NPR-07 binding to the free enzyme sterically hinders small substrate molecules from accessing the active site, consistent with a competitive inhibition model. In contrast, NPR-11 reduced Vmax while leaving Km unchanged, indicating that it does not interfere with substrate binding but instead impairs product release, characteristic of non-competitive inhibition. Interestingly, NPR-05 increased both Km and Vmax, indicating a more complex interaction mechanism with NA. This suggests that NPR-05 competes with the substrate when binding to the free enzyme, but once bound to the enzyme-substrate complex, it facilitates product release. This dual interaction leads to a substantial increase in enzymatic activity at higher substrate concentrations, distinguishing NPR-05 as a mixed-type inhibitor.

Using two mAbs, NPR-05 and NPR-07, which increased Km values in the kinetic MUNANA assay, we successfully isolated several escape mutants. In contrast, no true escape mutants emerged when using other mAbs (NPR-06, NPR-10, NPR-11, and NPR-12). While the inability to generate escape mutants with certain NA-targeting mAbs is well-documented^8,9,24^, this finding highlights the critical role of the NPR-05 and NPR-07 epitopes in viral replication. After escape selection, compensatory mutations in HA were observed, likely reducing HA avidity for sialic acid^39^. Indeed, when rescued on a wild-type HA backbone, most selected mutants exhibited significantly reduced replication efficiency in MDCK cells and impaired virion release from sialic acid.

While mAbs from different inhibitory categories (steric vs functional) may offer protection by blocking NA interaction with sialylated cellular receptors, functional inhibitors are expected to provide more durable immunity. As we showed, escape mutations occurring near functionally critical regions of NA can alter its enzymatic activity and disrupt the finely tuned balance between HA and NA functions. Consequently, mAbs that exert functional inhibition on NA may impose a greater evolutionary constraint on IAV escape, reducing the likelihood of their successful emergence through natural selection. Although this hypothesis requires further experimental validation, the kinetic MUNANA assay holds promise as a pre-screening tool for identifying mAbs most likely to drive the selection of escape mutants.

A particular limitation of the kinetic MUNANA assay is the need for complex calculations to transform raw data into meaningful results. This process requires determining initial reaction velocities and fitting the data to the non-linear Michaelis-Menten equation to estimate Km and Vmax parameters. To address this challenge, we developed a user-friendly Shiny web application that automates data processing and provides preliminary statistical analyses of mAb-induced effects on Km and Vmax. We also observed variability in the kinetic parameters across different batches of WT PR8 grown in eggs. While Vmax differences are expected as Vmax is dependent on virion amount, the Km changes are more challenging to explain. Still, different virus batches may be different in the chemical composition of allantoic fluid (cation content), may hold different amount of NA per virion or may have other unexplained differences. To account for such variability and isolate the true biological effects, a paired experimental design is recommended. However, when multiple batches are used within a single series of experiments, employing a mixed-effects model (with batch-specific random effects on Km and Vmax) can provide a statistically robust framework for drawing reliable conclusions.

Beyond computational complexity, the kinetic MUNANA assay has other limitations when assessing the properties of anti-NA antibodies. Firstly, while our study demonstrates that the assay can reveal Fab-mediated antiviral mechanisms, it does not determine whether targeting functionally critical NA epitopes also influences Fc-mediated protective functions. These include antibody-dependent cellular cytotoxicity, antibody-dependent phagocytosis, and complement activation. Previous studies^18–21^ have shown that broadly reactive or neutralizing anti-NA mAbs can mediate Fc-dependent effector functions, providing protection against IAV infection in mouse models. Secondly, the kinetic MUNANA assay generates interpretable results only for a subset of anti-NA mAbs—specifically, those that affect the functionality of NA’s active site. In our study, only three out of six tested mAbs altered NA functionality, which may not fully reflect the frequency of such anti-NA antibodies in a natural immune response. Finally, further work is needed to establish how different modes of NA inhibition by mAbs correlate with their protective efficacy in vivo.

Taken together, our findings suggest that mAbs increasing Km values in the kinetic MUNANA assay target functionally important NA epitopes near its active site. Mutations in these regions were associated with altered enzymatic activity, highlighting the critical role of these residues in the catalytic process. This supports the idea that mAbs directed against such sites may exert stronger antiviral effects. Our study provides a framework for using the kinetic MUNANA assay to identify distinct modes of NA inhibition and to guide future investigations into how individual mAbs confer protective immunity by targeting functionally relevant epitopes.

## Methods

### Ethical approvals

The study and all animal procedures were performed according to relevant guidelines and regulations and following protocols approved under ethical permits 1666/19 and 38/23 granted by the Gothenburg Regional Animal Ethics Committee.

### rNA design and purification

rNA was designed as a fusion protein comprising a self-tetramerizing tetrabrachion sequence^28,29^ and the extracellular domain of NA from the PR8 virus (GenBank: AB671290.1), linked by a short GT motif. A *Gaussia sp*. signal peptide was added prior the main encoding sequence of the construct to direct it to the secretion pathway^40^. For purification and enzymatic biotin labeling, an 8×His tag and Avi-tag were included between the signal peptide and tetrabrachion sequence.

The rNA sequence, codon-optimized using the jcat tool (http://www.jcat.de/), was synthesized by Eurofins and inserted into the pCEP4 expression vector. rNA was expressed in the Expi293 Expression System (ThermoFisher, cat. #A14635) and purified via its His-tag using a Ni-Sepharose excel column (Cytiva, cat. #17371205) on an ÄKTA Start system.

### Development of a stable NA-expressing cell line

A stable cell line was generated using murine fibroblast NCTC929 cells. A fusion construct encoding EGFP and full-length NA from the PR8 virus (EGFPNA) was cloned into the pQCXIP vector. Retroviral packaging Platinum E cells were transfected with the construct using the calcium-phosphate method^41,42^. After four rounds of transduction, NA-expressing NCTC929 cells (NCTC-EGFP) were selected with puromycin (10 µg/ml). NA-positive cells, identified by green fluorescence, were enriched using FACS Aria III to isolate a population with high NA expression.

### Detection of anti-NA Abs in ELISA

Three ELISA formats were used to detect anti-NA Abs: two indirect ELISAs (on rNA or PR8 virus adsorbed on the solid phase) and a cell-based ELISA. For the indirect assays, Nunc Maxisorp 96-well plates (ThermoFisher, cat. #442404) were coated with either rNA (0.5 µg/ml) or PR8 virus in PBS. For the cell-based ELISA, NA-expressing NCTC929 cells were grown to confluence in 96-well plates, fixed with 0.025% glutaraldehyde (Sigma Aldrich, cat. #G6257) for 5 min and blocked with 0.2% gelatin in PBS. Bound Ig was detected using anti-mouse Ig HRP-conjugated Abs (Vector Laboratories, cat. #PI-2000-1). Tris-buffered saline (TBS, pH 7.4) with Tween-20 was used as the working buffer.

For the competitive ELISA, mAbs were biotinylated using EZ-Link NHS-Biotin (Thermo Fisher, cat. #20217). Plates were coated with with chicken egg allantoic fluid containing PR8 in PBS (1:200 dilution). Biotin labelled mAb working concentration was determined by running competitive ELISA with itself and choosing the concentration that gives the highest OD value in the absence of unlabelled mAb but low OD value in the presence of the unlabelled mAb. Biotin-labeled and unlabeled mAbs (20 µg/ml) were co-incubated on PR8-coated plates at RT for 2 h. Streptavidin-HRP (Thermo Fisher, cat. #SA10001) was added and incubated for 30 min at RT. PBS + 0.05% Tween-20 (PBS-T) was used as the working buffer.

For confirmation of escape mutants, indirect ELISA with UV inactivated WT and mutant virus were used to coat the plates (1:50 dilution) in PBS. mAbs at 1.25 μg/ml in PBS-T were tested against the different virus, with an anti-NA mAb, NA-1C1, was used as a positive control^31,43^. Anti-mouse IgG HRP-conjugated Abs (Vector Laboratories, cat. #PI-2000-1) was used to detect bound IgG.

In all assays plates were developed with TMB substrate for 5–7 min, and the reaction was stopped by adding 2 M H_2_SO_4_.

### ELLA

96-well plates were coated with fetuin at 25 μg/ml (Sigma Aldrich, cat. #F2379). To determine IC_50_ values, mAbs and PR8 virus were pre-incubated at +37 °C for 1 h before being transferred to the fetuin-coated plates. Proper virus titre was determined using the same assay, where the OD is 2x the background OD. MAbs concentration used were 25 μg/ml, apart form NPR-10 which started at 2.7 μg/ml, then diluted 3-fold. For enzymatic activity comparisons of PR8 mutants, normalized virus amounts were directly added to the wells. The plates were incubated at +37 °C for 20 h. Virus incubation and mAb dilution were done in 1× MUNANA buffer with 0.05% Tween 20, while PBS was used for lectin-HRP incubation. An anti-SARS-CoV-2 mAb was used as a control, mAb #27^44^.

ELLA was also used to confirm the escape mutants. The assay was carried out similarly to the methods explained above but with some changes. Briefly, WT and mutant viruses (1:12.5 dilution) were pre-incubated with mAbs (10 μg/ml) for 1 h at +37 °C. Virus and mAb solution were transferred to fetuin coated plates and incubated for 20 h at +37 °C.

For characterization of escape mutants, the same assay was carried out with the absence of mAbs. The amount of virus added was normalized according to number of virions, starting at 120,000 then serially diluted two-fold.

All assay plates were detected using peanut lectin-HRP at 0.1 μg/ml (Sigma Aldrich, cat. #L7759), incubated at +37 °C for 2 h. Plates were developed with TMB substrate for 10–15 min, and the reaction was stopped by adding 2 M H_2_SO_4_.

### Mice

BALB/c mice were purchased from Janvier labs, France. They were housed in the specific pathogen free animal facility of Experimental Biomedicine Unit (EBM) at the University of Gothenburg under 12 h light, 12 h dark cycle. Female mice eight to twelve weeks old were used in the experiments.

### mAb development and purification

Balb/c mice were primed intranasally with PR8 virus (50 TCID_50_) under isofluorane anesthesia and boosted intraperitoneally (i.p.) with 5 U of rNA 67–97 d later. Two and three days prior to fusion, each mouse received additional 2.5 U of rNA i.p. Four immunization campaigns were conducted and spleens from three mice with highest Ab titers were used for hybridoma production (Supplementary Fig. 1C). Mice were euthanized by cervical dislocation, while under isofluorane anesthesia. Murine splenocytes were fused with SP2/m-IL6 myeloma cells using polyethylene glycol (Sigma, cat. #P7181). Hybridomas were selected in HAT (Sigma, cat. #H0262) and HT (Sigma, cat. #H0137) media following standard protocols.

Hybridoma cells were cultured in serum-free medium (ThermoFisher, cat. #12045076), and their filtered supernatants were used for mAb isolation using a HiTrap protein G column (Cytiva, cat. #17040501) on an ÄKTA Start system.

### Kinetic MUNANA assay

The MUNANA assay was performed using 2× MUNANA buffer (66.6 mM MES, 8 mM CaCl_2_, pH 6.5, stored at +4 °C). A 4-MU standard solution (6.4 mM) was prepared by dissolving 4-methylumbelliferone (Sigma, cat. #M1381) in absolute ethanol, followed by the addition of 0.9% NaCl. For the standard dilution series, 4-MU was titrated in MUNANA buffer from 50 to 0.78 μM.

PR8 virus (4×) and mAb (4×) solutions in MUNANA buffer were prepared in round-bottom 96-well plates (Sarstedt, cat. #83.3925). A 2× substrate dilution series (from 2000 to 15.6 μM) was prepared, with 50 μL of each dilution added to black assay plates (Greiner Bio-One, cat. #655076). All plates were warmed to +37 °C for 15 min before the assay. Next, 25 μL of mAb solution and 25 μL of PR8 virus solution were transferred to the assay plates using a multichannel pipette and mixed gently. All mAbs were tested at an empirically chosen final concentration of 10 µg/ml. The assay was incubated at +37 °C for 1 h and 15 min, with optical data collected every 1.5 min (6 flashes per read) using a SpectraMax i3x Microplate Reader. Samples and standards were excited by ultraviolet light with wavelength 355 ± 9 nm and its emission was detected at 454 ± 30 nm. An anti-HA mAb, H2-6A1, was used as a control for this assay^45^.

### Data processing and statistical inference in the kinetic MUNANA assay

**Fig. 5:**
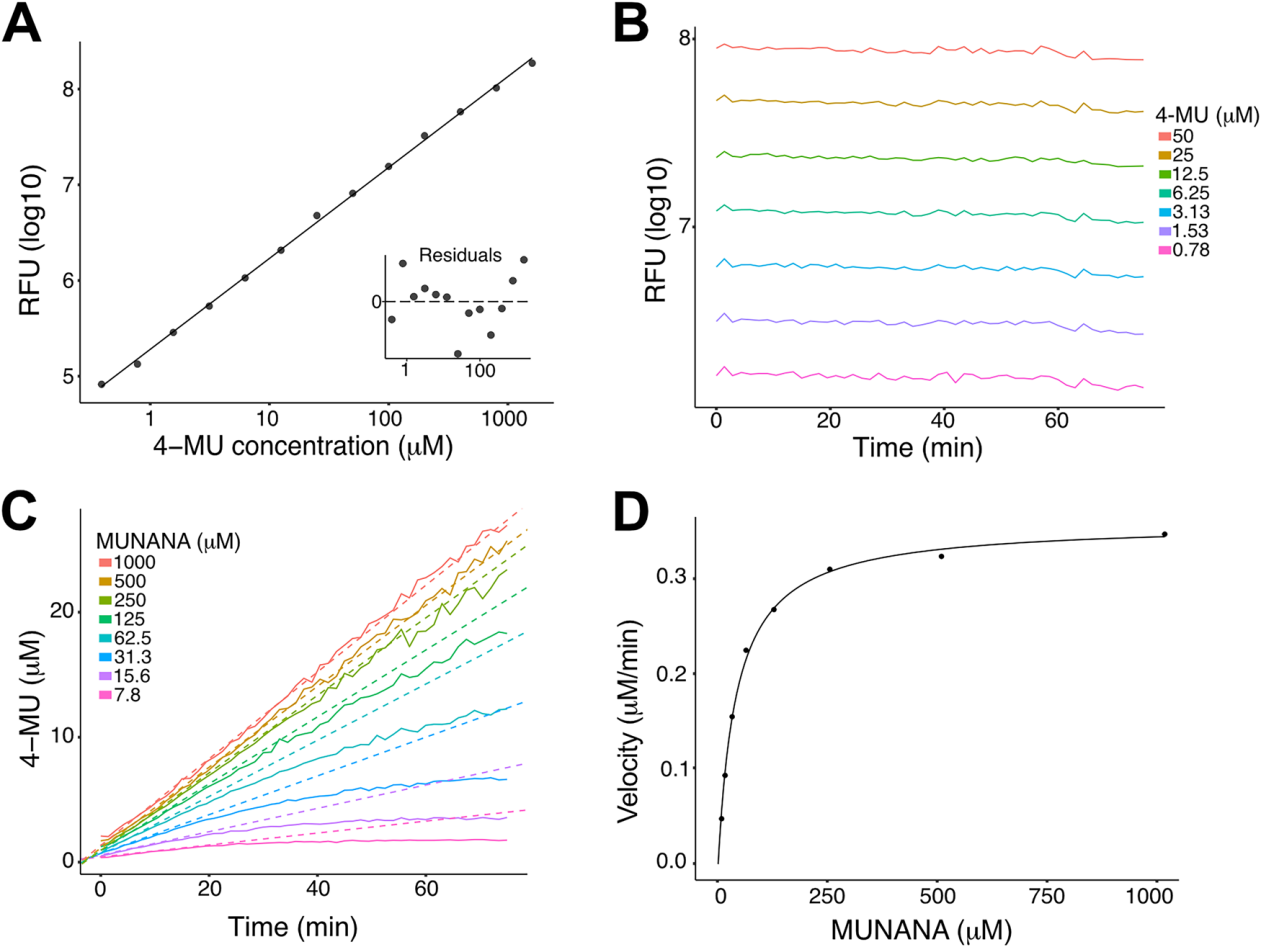
Processing of kinetic MUNANA assay data. **A** Linear regression model applied to fluorescence data after log transformation of concentration and fluorescence values. Residuals plotted against fitted data are shown in the subplot. **B** Gradual decay of fluorescence in the standard titration series observed during the experiment. **C** Progress curves (solid lines) with fitted initial velocities (dashed lines). **D** Fitted Michaelis-Menten curve based on experimental data.

To convert optical data into 4-MU concentrations, serial dilutions of 4-MU with known concentrations were used as standards (Fig. 5A). Since the fluorescence of 4-MU gradually decreased due to photobleaching and temperature effects (Fig. 5B), standard curve parameters were recalculated at each assay time point to ensure accurate conversion of optical data into 4-MU concentrations. Progress curves of 4-MU accumulation were modeled using quadratic linear regression (*y = ax*^*2*^ *+ bx* + *c*) if *a* coefficient was both positive and statistically significant (*p* < 0.05). In cases where these conditions were not met, ordinary linear regression (*y = bx* + *c*) was applied. The *b* coefficient was used as an estimate of the initial reaction velocity (Fig. 5C). Reaction velocities were then plotted against substrate concentrations (Fig. 5D) and fitted non-linearly to determine the Vmax and Km coefficients of the Michaelis-Menten equation. The 95% confidence intervals for Km and Vmax were derived from the regression model, following the methodology described by Ruckstuhl^46^.

In each assay, an IgG2a mAb that does not bind to NA (or wild-type PR8 virus) was used as a control. The statistical significance of deviations in Km and Vmax caused by mAb (or mutation in NA) from their levels in the control sample was assessed using a modified Michaelis-Menten equation(46):

$$\upsilon =\frac{{(V}_{\max }+{zV})\times [S]}{{K}_{m}+{zK}+[S]}$$

where, *Vmax*, maximum reaction velocity reachable in a reference sample, *V*, change of the Vmax value in the sample of interest, *Km*, Michaelis constant of enzyme in control sample, *K*, change of the Michaelis constant in the presence of mAb or due to mutation, z, logical indicator showing if mAb added or mutation present (*z* = 1) or not (*z* = 0).

To evaluate statistical significance from multiple technical replicates, three methods were employed, each with distinct advantages and limitations:

1. Fisher’s Method:
  *P*-values from technical replicates were combined using Fisher’s method^47^. This approach provides a straightforward summary of statistical significance but does not allow for direct estimation of means or 95% CI for Vmax and Km.
2. ANOVA:
  ANOVA enables the calculation of means and 95% CIs for Vmax and Km, but it is less sensitive for detecting statistical differences between the inhibitory effects of mAbs or the enzymatic activity of virus mutants.
3. Non-Linear Mixed-Effect Model (NLME):

Michaelis-Menten curves from technical replicates were fitted using an NLME model, with Vmax and Km treated as both fixed and random effects. Fixed effects varied depending on the inhibitors used, while random effects accounted for variation among experimental replicates. NLME is the most robust method, providing means, 95% CIs, and *p*-values. However, it may fail to converge under high data noise or exhibit residual heteroscedasticity.

*P*-values obtained using all three methods are provided for reference.

### Kinetic MUNANA app

We developed an open-access application for processing kinetic MUNANA assay data, utilizing the core functionality of R (version 3.6.1). The graphical user interface was built using the shiny package (version 1.6.0), with all plots generated by the ggplot2 package (version 3.3.5). Word reports are created automatically using the officer package (version 0.3.18).

### Escape virus selection

Wild-type PR8 virus (0.01 MOI) was pre-incubated with mAbs for 1 h at RT and then added to a confluent monolayer of MDCK cells. The plates were incubated for 3 d, after which the supernatants were collected. For subsequent passages, the supernatants were diluted 1:10 in cell culture medium before being used for the next round of selection. At each passage, the mAb concentration was doubled, starting from 0.25 IC_50_ as determined by ELLA.

Viral RNA was extracted from the supernatants using the Zymo Quick Viral RNA 96 kit (cat. #R1041). cDNA was generated by reverse transcription and amplified using Q5 polymerase (NEB, cat. #M049L). PCR products including NA and HA sequences were purified and sequenced.

DMEM + 1 μg/ml TPCK- trypsin (BioNordika, cat.# LS003740) + 10 µg/ml gentamicin (Thermo Fisher, cat.# 15710064) was the cell culture medium used for this assay.

Note that for identifying the mutations, we used the numbering system where the methionine amino acid of a full length mature protein is in position 1. Functional sites and mAb-binding sites were highlighted on 3D models of NA’s head using the 3dproteinimaging web-site^48^.

### Virus rescue by reverse genetics

Mutant NA plasmids were generated by performing site directed mutagenesis on WT NA plasmid using KOD Xtreme Hot Start DNA Polymerase (Novagen, cat.# 71975-3) following manufacturer’s protocol. The day before transfection, HEK293T and MDCK cells were seeded at 1:1 ratio for a total of 250,000 cells/2 mL per well in 6-well plates (Sarstedt, cat.# 83.3920.300). The next day, cells were transfected with 1ug/IAV gene plasmid (mutant NA and WT HA, NP, NS1, M, PA, PB1, PB2), a total of 8 genes, using Lipofectamine 2000 transfection reagent at 1ul/plasmid (Invitrogen, cat.# 11668-019) in Opti-MEM reduced serum medium (Gibco, cat.# 31985-062), according to manufacturer’s instructions. 1 mL of DMEM + 10% FBS + 10ug/ml gentamicin was added to each transfection mix and added to the wells. 24 h post transfection, wells were washed gently with PBS and the media was changed to DMEM + 0.3% bovine serum albumin (BSA, Sigma Aldrich, cat.# A7906) + 1 μg/ml TPCK-Trypsin + 10 μg/ml gentamicin at 2 mL/well. 48 h post transfection, a new plate was seeded with the same number of cells and allowed to grow to 80–90% confluency. The day after, the original plate was spun down at 300 G for 5 min and 200 μl of supernatants were collected per well and used to infect the plate then incubated for 1 h incubation at +37 °C with 5% CO_2_. After the incubation, DMEM + 0.3% BSA + 1 μg/ml TPCK-Trypsin + 10 μg/ml gentamicin was added to each well to a total volume of 2 ml/well and kept in the +37 °C with 5% CO_2_ for 2–3 days. Monolayer was checked for cytopathic effect before changing plates, which is a sign that the virus is growing and infecting the cells. HA and NA sequence was verified by Sanger sequencing. Hemagglutination assay was used to confirm that the rescued viruses are functional. After, TCID50 assay was used to determine the viral titre.

### Plaque reduction assay

MDCK cells were seeded at 400,000 cells/mL in 12-well plates the day before the assay. The next day, the cells were infected with 400 μl of 25PFU per virus in plaque assay media (DMEM with glutamine + 1 M HEPES (Gibco, cat.# 11560496) + 10 mM Sodium Pyruvate (Gibco, cat.# 11360070) + 10 μg/ml gentamicin). Plates were incubated for 1 h in +37 °C supplemented with 5% CO_2_, with rocking every 15 min. The inoculum was aspirated and 1 mL of 1.2% Avicel (Sigma Aldrich, cat.# 435244) in MEM media with 0.1 μg/ml TPCK-trypsin overlay + 1 μg/ml mAb was added per well after the incubation and incubated for 3 days at +37 °C supplemented with 5% CO_2_. The overlay was removed, then the cells were washed 1x with PBS and were fixed with 3.7% paraformaldehyde (PFA, Thermo Fisher, cat.# 28908) in PBS for 30 min at +4 °C. PFA solution was aspirated followed by the addition of 0.5% crystal violet for staining and visualization. An anti-SARS-CoV-2 mAb was used as a control, mAb #27^44^.

### HA-based virus normalization

Wild-type and mutant viruses were normalized using a hemagglutination assay. Viruses were serially diluted in PBS, and chicken red blood cells (Hatunalab AB) were added to each dilution. The mixtures were incubated for 30 min RT, and viral titers were subsequently recorded.

To standardize the number of viral particles for subsequent experiments, the following formula was used:

$$N=T\times \frac{{C}_{{RBC}}}{V}$$

where, *N*, estimated number of viral particles, *T*, virus titer, *C*_*RBC*_, concentration of red blood cells, *V*, volume of the virus dilution.

### RBC virus release assay

To assess the relative activity of NA in the wildtype and mutant virus, we performed an RBC virus release assay^49^. Normalized virus amounts were serially diluted in PBS containing CaCl_2_ and MgCl_2_ and added to 96-well plates. To each well, 1% chicken RBCs were added. The plates were initially incubated at +4 °C for 1 h to determine the starting HA titer. Following this, the plates were incubated at +37 °C, and HA titers were measured every 30 min over 7 h. The results are expressed as the percentage of elution relative to the wild-type PR8 virus titer at the end of the incubation period.

### Virus growth kinetics in MDCK cells

MDCK cells were seeded in a 96 well plate a day before the assay. An MOI of 0.01 was used for all wild-type and mutant viruses. Supernatants were collected at 18-, 24-, 36- and 72 h post infection and the virus in the supernatant was measured by TCID_50_ in MDCK cells.

### Statistics

GraphPad Prism was used to conduct statistical analysis on Figs. 2A and 4. In Fig. 2A, to calculate the IC_50_ value for the mAbs, logarithmic non-linear regression was used. In Fig. 4D, Two-way ANOVA with multiple comparisons and Dunnett testing at an alpha of 0.05 was performed to calculate the statistical differences in viral growth kinetics. In Fig. 4E, One-way ANOVA with multiple comparisons and Dunnett testing at an alpha of 0.05 was performed to calculate the statistical differences on the areas under the curve of the data. * denotes a *p*-value of ≤0.05, ** denotes a *p*-value of ≤0.01, *** denotes a *p*-value of ≤0.001, **** denotes a *p*-value of ≤0.0001.

## Supplementary information

SUPPLEMENTARY MATERIAL

## Data Availability

Data underlying the results is present within the paper or its supplementary information.

## Code availability

The source code for the app is available on GitHub (https://github.com/ilya-v-smirnov/Kinetic-MUNANA-App). The app is available at https://angeletti-lab.shinyapps.io/kinetic-munana-app/.

## References

[1] Dunning, J., Thwaites, R. S. & Openshaw, P. J. M. Seasonal and pandemic influenza: 100 years of progress, still much to learn. *Mucosal. Immunol.* **13**, 566–573 (2020). 32317736 10.1038/s41385-020-0287-5 PMC7223327

[2] Wu, N. C. & Ellebedy, A. H. Targeting neuraminidase: the next frontier for broadly protective influenza vaccines. *Trends Immunol.* **45**, 11–19 (2024). 38103991 10.1016/j.it.2023.11.001 PMC10841738

[3] Su, H. C., Feng, I. J., Tang, H. J., Shih, M. F. & Hua, Y. M. Comparative effectiveness of neuraminidase inhibitors in patients with influenza: a systematic review and network meta-analysis. *J. Infect. Chemother.* **28**, 158–169 (2022). 34840038 10.1016/j.jiac.2021.11.014

[4] Heneghan, C. J. et al. Neuraminidase inhibitors for influenza: a systematic review and meta-analysis of regulatory and mortality data. *Health Technol. Assess.* **20**, 1–242 (2016). 27246259 10.3310/hta20420 PMC4904189

[5] Krammer, F. et al. Influenza. *Nat. Rev. Dis. Prim.* **4**, 1–21 (2018). 29955068 10.1038/s41572-018-0002-y PMC7097467

[6] Wu, N. C. & Wilson, I. A. Influenza hemagglutinin structures and antibody recognition. *Cold Spring Harb. Perspect. Med.* **10**, a038778 (2020). 31871236 10.1101/cshperspect.a038778 PMC7397844

[7] Sakai, T., Nishimura, S. I., Naito, T. & Saito, M. Influenza A virus hemagglutinin and neuraminidase act as novel motile machinery. *Sci. Rep.* **7**, 45043 (2017). 28344335 10.1038/srep45043 PMC5366856

[8] McAuley, J. L., Gilbertson, B. P., Trifkovic, S., Brown, L. E. & McKimm-Breschkin, J. L. Influenza virus neuraminidase structure and functions. *Front. Microbiol.* **10**. Available from: https://www.frontiersin.org/articles/10.3389/fmicb.2019.00039 (2019). 10.3389/fmicb.2019.00039 PMC6362415 30761095

[9] Cohen, M. et al. Influenza A penetrates host mucus by cleaving sialic acids with neuraminidase. *Virol. J.* **10**, 321 (2013). 24261589 10.1186/1743-422X-10-321 PMC3842836

[10] de Vries, E., Du, W., Guo, H. & de Haan, C. A. M. Influenza A virus hemagglutinin–neuraminidase–receptor balance: preserving virus motility. *Trends Microbiol.* **28**, 57–67 (2020). 31629602 10.1016/j.tim.2019.08.010 PMC7172302

[11] Smet, A. et al. Antibodies directed towards neuraminidase restrict influenza virus replication in primary human bronchial epithelial cells. *PLOS ONE* **17**, e0262873 (2022). 35100294 10.1371/journal.pone.0262873 PMC8803191

[12] Gao, J. et al. Antigenic drift of the Influenza A(H1N1)pdm09 virus neuraminidase results in reduced effectiveness of A/California/7/2009 (H1N1pdm09)-specific antibodies. *mBio* **10**, e00307–19 (2019). 30967460 10.1128/mBio.00307-19 PMC6456748

[13] Couch, R. B. et al. Antibody correlates and predictors of immunity to naturally occurring influenza in humans and the importance of antibody to the neuraminidase. *J. Infect. Dis.* **207**, 974–981 (2013). 23307936 10.1093/infdis/jis935 PMC3633450

[14] Maier, H. E. et al. Pre-existing antineuraminidase antibodies are associated with shortened duration of Influenza A(H1N1)pdm virus shedding and illness in naturally infected adults. *Clin. Infect. Dis.* **70**, 2290–2297 (2020). 31300819 10.1093/cid/ciz639 PMC7245146

[15] Rosu, M. E. et al. Contribution of neuraminidase to the efficacy of seasonal split influenza vaccines in the Ferret model. *J. Virol.* **96**, e01959-21 (2022). 10.1128/jvi.01959-21 PMC8941921 35107371

[16] Westgeest, K. B. et al. Genetic evolution of the neuraminidase of Influenza A (H3N2) viruses from 1968 to 2009 and its correspondence to haemagglutinin evolution. *J. Gen. Virol.* **93**, 1996–2007 (2012). 22718569 10.1099/vir.0.043059-0 PMC3542130

[17] Sandbulte, M. R. et al. Discordant antigenic drift of neuraminidase and hemagglutinin in H1N1 and H3N2 influenza viruses. *Proc. Natl. Acad. Sci. USA* **108**, 20748–20753 (2011). 22143798 10.1073/pnas.1113801108 PMC3251064

[18] DiLillo, D. J., Palese, P., Wilson, P. C. & Ravetch, J. V. Broadly neutralizing anti-influenza antibodies require Fc receptor engagement for in vivo protection. *J. Clin. Invest.* **126**, 605–610 (2016). 10.1172/JCI84428 PMC4731186 26731473

[19] Stadlbauer, D. et al. Broadly protective human antibodies that target the active site of influenza virus neuraminidase. *Science* **366**, 499–504 (2019). 31649200 10.1126/science.aay0678 PMC7105897

[20] Lei, R. et al. Leveraging vaccination-induced protective antibodies to define conserved epitopes on influenza N2 neuraminidase. *Immunity* **56**, 2621–2634.e6 (2023). 37967533 10.1016/j.immuni.2023.10.005 PMC10655865

[21] Job, E. R. et al. Fcγ receptors contribute to the antiviral properties of influenza virus neuraminidase-specific antibodies. *mBio* **10**, 10.1128/mbio.01667-19 (2019). 10.1128/mBio.01667-19 PMC6805988 31641082

[22] Momont, C. et al. A pan-influenza antibody inhibiting neuraminidase via receptor mimicry. *Nature* **618**, 590–597 (2023). 37258672 10.1038/s41586-023-06136-y PMC10266979

[23] Memoli, M. J. et al. Evaluation of antihemagglutinin and antineuraminidase antibodies as correlates of protection in an Influenza A/H1N1 virus healthy human challenge model. *mBio* **7**, e00417-16 (2016). 27094330 10.1128/mBio.00417-16 PMC4959521

[24] Krammer, F. et al. NAction! how can neuraminidase-based immunity contribute to better influenza virus vaccines?. *mBio* **9**, e02332-17 (2018). 29615508 10.1128/mBio.02332-17 PMC5885027

[25] Gao, J., Couzens, L. & Eichelberger, M. C. Measuring influenza neuraminidase inhibition antibody titers by enzyme-linked lectin assay. *J. Vis. Exp.* **115**, 54573 (2016). 10.3791/54573 PMC5091984 27684188

[26] Leang, S. K. & Hurt, A. C. Fluorescence-based neuraminidase inhibition assay to assess the susceptibility of influenza viruses to the neuraminidase inhibitor class of antivirals. *J. Vis. Exp.* **122**, 55570 (2017). 10.3791/55570 PMC5564701 28448045

[27] Marathe, B. M., Lévêque, V., Klumpp, K., Webster, R. G. & Govorkova, E. A. Determination of neuraminidase kinetic constants using whole influenza virus preparations and correction for spectroscopic interference by a fluorogenic substrate. *PLoS One* **8**, e71401 (2013). 23977037 10.1371/journal.pone.0071401 PMC3744557

[28] Stetefeld, J. et al. Crystal structure of a naturally occurring parallel right-handed coiled coil tetramer. *Nat. Struct. Biol.* **7**, 772–776 (2000). 10966648 10.1038/79006

[29] Streltsov, V. A., Schmidt, P. M. & McKimm-Breschkin, J. L. Structure of an Influenza A virus N9 neuraminidase with a tetrabrachion-domain stalk. *Acta Crystallogr F. Struct. Biol. Commun.* **75**, 89–97 (2019). 30713159 10.1107/S2053230X18017892 PMC6360442

[30] Gubareva, L. & Mohan, T. Antivirals targeting the neuraminidase. *Cold Spring Harb. Perspect. Med.* **12**, a038455 (2022). 32152244 10.1101/cshperspect.a038455 PMC8725622

[31] Kosik, I. & Yewdell, J. W. Influenza A virus hemagglutinin specific antibodies interfere with virion neuraminidase activity via two distinct mechanisms. *Virology* **500**, 178–183 (2017). 27825034 10.1016/j.virol.2016.10.024 PMC5127735

[32] Gaymard, A., Briand, N. L., Frobert, E., Lina, B. & Escuret, V. Functional balance between neuraminidase and haemagglutinin in influenza viruses. *Clin. Microbiol. Infect.* **22**, 975–983 (2016). 27424943 10.1016/j.cmi.2016.07.007

[33] Myers, J. L. et al. Compensatory hemagglutinin mutations alter antigenic properties of influenza viruses. *J. Virol.* **87**, 11168–11172 (2013). 23926344 10.1128/JVI.01414-13 PMC3807274

[34] Silverstein, T. P. When both Km and Vmax are altered, Is the enzyme inhibited or activated?. *Biochem. Mol. Biol. Educ.* **47**, 446–449 (2019). 30908872 10.1002/bmb.21235

[35] Turberville, A., Semple, H., Davies, G., Ivanov, D. & Holdgate, G. A. A perspective on the discovery of enzyme activators. *SLAS Discov.* **27**, 419–427 (2022). 36089246 10.1016/j.slasd.2022.09.001

[36] Pesaresi, A. Mixed and non-competitive enzyme inhibition: underlying mechanisms and mechanistic irrelevance of the formal two-site model. *J. Enzym. Inhibit. Med. Chem.* **38**, 2245168 (2023). 10.1080/14756366.2023.2245168 PMC10683834 37577806

[37] Job, E. R. et al. Antibodies directed toward neuraminidase N1 control disease in a mouse model of influenza. *J. Virol.* **92**, e01584-17 (2018). 29167342 10.1128/JVI.01584-17 PMC5790960

[38] Rijal, P. et al. Broadly inhibiting antineuraminidase monoclonal antibodies induced by trivalent influenza vaccine and H7N9 infection in humans. *J. Virol.* **94**, 10.1128/jvi.01182-19 (2020). 10.1128/JVI.01182-19 PMC6997757 31748388

[39] McKimm-Breschkin, J. L. Resistance of influenza viruses to neuraminidase inhibitors — a review. *Antivir. Res.* **47**, 1–17 (2000). 10930642 10.1016/s0166-3542(00)00103-0

[40] Stern, B., Olsen, L. C., Trösse, C., Ravneberg, H. & Pryme, I. F. Improving mammalian cell factories: the selection of signal peptide has a major impact on recombinant protein synthesis and secretion in mammalian cells. *Trends. Cell. Mol. Biol.* **2**, 1–17 (2007).

[41] Morita, S., Kojima, T. & Kitamura, T. Plat-E: an efficient and stable system for transient packaging of retroviruses. *Gene Ther.* **7**, 1063–1066 (2000). 10871756 10.1038/sj.gt.3301206

[42] Jordan, M., Schallhorn, A. & Wurm, F. M. Transfecting mammalian cells: optimization of critical parameters affecting calcium-phosphate precipitate formation. *Nucleic Acids Res.* **24**, 596–601 (1996). 8604299 10.1093/nar/24.4.596 PMC145683

[43] Yewdell, J. W., Frank, E. & Gerhard, W. Expression of Influenza A virus internal antigens on the surface of infected P815 cells. *J. Immunol.* **126**, 1814–1819 (1981). 7217668

[44] Scharf, L. et al. Longitudinal single-cell analysis of SARS-CoV-2–reactive B cells uncovers persistence of early-formed, antigen-specific clones. *JCI Insight* 8(1). Available from: https://insight.jci.org/articles/view/165299 (2023). 10.1172/jci.insight.165299 PMC9870078 36445762

[45] Gerhard, W., Yewdell, J., Frankel, M. E. & Webster, R. Antigenic structure of influenza virus haemagglutinin defined by hybridoma antibodies. *Nature* **290**, 713–717 (1981). 6163993 10.1038/290713a0

[46] Ruckstuhl, A. Introduction to Nonlinear Regression. In IDP Institut fur Datenanalyse und Prozessdesign, Zurcher Hochschule fur Angewandte Wissenschaften. p. 365. Available online: https://stat.ethz.ch/~stahel/courses/cheming/nlreg10E.pdf (2010).

[47] Fisher, R. A. Theory of statistical estimation. *Math. Proc. Camb. Philos. Soc.* **22**, 700–725 (1925).

[48] Tomasello, G., Armenia, I. & Molla, G. The Protein Imager: a full-featured online molecular viewer interface with server-side HQ-rendering capabilities. *Bioinformatics* **36**, 2909–2911 (2020). 31930403 10.1093/bioinformatics/btaa009

[49] Kosik, I. et al. Neuraminidase inhibition contributes to Influenza A virus neutralization by anti-hemagglutinin stem antibodies. *J. Exp. Med.* **216**, 304–316 (2019). 30683737 10.1084/jem.20181624 PMC6363425

