# Supplementary material for: Kinetic MUNANA assay reveals functionally relevant antibody epitopes on Influenza A virus neuraminidase
Source: Npj Viruses. 2025 May 10;3:40. doi: 10.1038/s44298-025-00123-y (PMC12065816; doi:10.1038/s44298-025-00123-y)
Supplement: Supplementary file 1 — SUPPLEMENTARY MATERIAL [file 44298_2025_123_MOESM1_ESM.pdf]

SUPPLEMENTARY MATERIAL

Kinetic MUNANA Assay Reveals Functionally Relevant Antibody Epitopes on Influenza A Virus Neuraminidase

Ilya V. Smirnov[1,,#], Danica F. Besavilla[1,*], Karin Schön[1], Hannes Axelsson[1], Davide Angeletti[1,2,#]*

[1]Department of Microbiology and Immunology, Institute of Biomedicine, University of Gothenburg, Gothenburg, Sweden

[2]SciLifeLab, Institute of Biomedicine, University of Gothenburg, Gothenburg, Sweden

[*]Equal contribution

[#]Correspondence: smirnov.iv.mail@gmail.com, (I.V.S.), davide.angeletti@gu.se (D.A.)

Supplementary Tables

Supplementary Table 1. P-values calculated by different statistical method estimating effects of anti-NA mAbs on kinetic parameters of NA function.

Parameter	mAb	Method	P-value	Significance stars
Vmax	NPR-05	Fisher's	< 0.0001	***
		ANOVA	< 0.0001	***
		NLME	< 0.0001	***
	NPR-06	Fisher's	0.16	
		ANOVA	0.58	
		NLME	0.093	
	NPR-07	Fisher's	1	
		ANOVA	0.82	
		NLME	0.23	
	NPR-10	Fisher's	0.88	
		ANOVA	0.75	
		NLME	0.57	
	NPR-11	Fisher's	0.0002	***
		ANOVA	0.27	
		NLME	0.011	*
	NPR-12	Fisher's	0.26	
		ANOVA	0.76	
		NLME	0.21	
Km	NPR-05	Fisher's	0.0001	***
		ANOVA	0.0003	***
		NLME	< 0.0001	***
	NPR-06	Fisher's	1	
		ANOVA	0.91	
		NLME	0.83	
	NPR-07	Fisher's	0.084	
		ANOVA	0.0009	***
		NLME	0.0024	**
	NPR-10	Fisher's	0.92	
		ANOVA	0.15	
		NLME	0.16	
	NPR-11	Fisher's	1	
		ANOVA	0.29	
		NLME	0.45	
	NPR-12	Fisher's	1	
		ANOVA	0.77	
		NLME	0.50	

Supplementary Table 2. Conversion of optical density values obtained in cell ELISA or indirect ELISA on rNA or PR8 virus to a rank scale.

	**Cell ELISA**	**rNA**	**PR8**
Very High	>2.0	>3.0	>2.5
High	1.5-2.0	2.0-3.0	1.3-2.5
Moderate	1.0-1.5	1.0-2.0	0.7-1.3
Weak	0.3-1.0	0.2-1.0	0.2-0.7
No	<0.3	<0.2	<0.2

Supplementary Figures

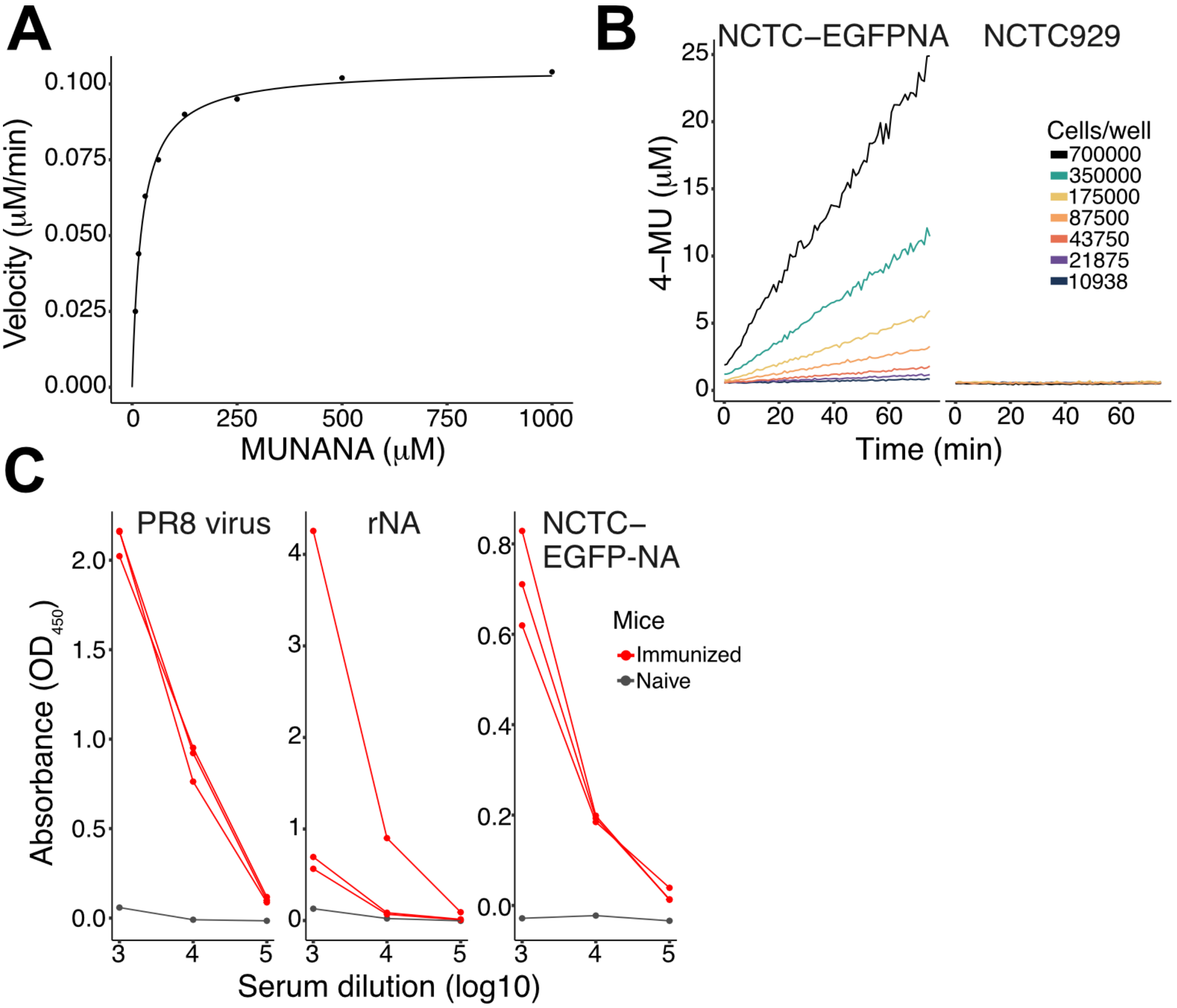

Supplementary Figure 1. Confirmation of enzymatic activity of soluble rNA and stable cell line expressing NA coupled with EGFP (EGFPNA) in kinetic MUNANA assay. **A**. Enzymatic activity of rNA, Vmax = 0.105 μM/min, Km = 22.72 μM. **B.** NCTC-EGFPNA cells demonstrate sialidase activity in MUNANA assay in contrast to wild-type cells. Experiment conducted with constant MUNANA concentration (150 μM) and variable number of cells in wells. **C.** Detection of anti-NA antibodies in sera of immunized mice used for hybridoma fusion by ELISA on PR8 virus, fixed NCTC-EGFPNA cells or adsorbed rNA. Each line correspond to an individual mice (red-immunized; black-naïve).

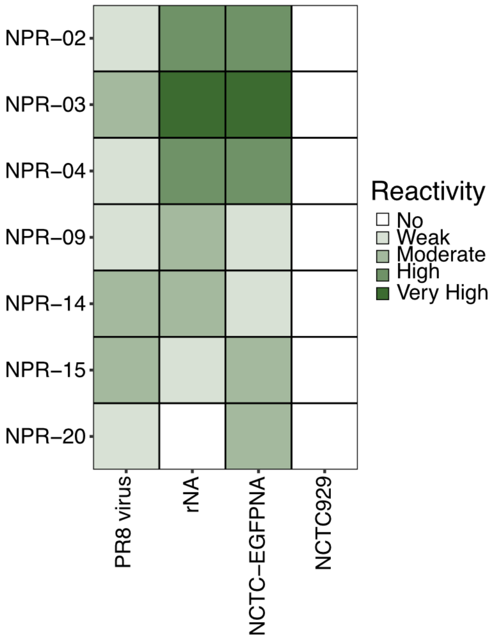

Supplementary Figure 2. Immunoreactivity of IgM (NPR-02, -03, -04, -09, -14, -20) and IgA (NPR-15) mAbs in ELISA with NA from various sources: adsorbed PR8 virus particles, recombinant protein, or expressed on the membranes of NCTC929 cells as an EGFP fusion protein. Reactivity with intact cells is shown as a specificity control. A relative scale is used, as the assays have different sensitivities (Supplementary Table 2).

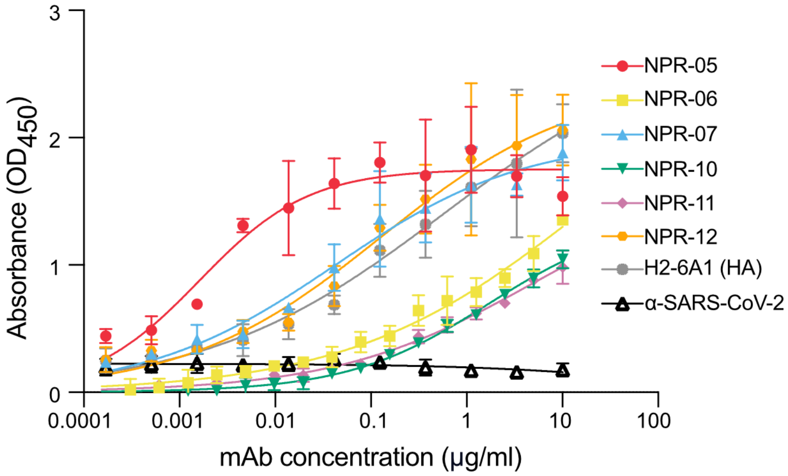

Supplementary Figure 3. ELISA curves showing IgG2a mAbs binding to coated UV-inactivated PR8 virions. Data are shown as mean ± SEM, (N = 2).

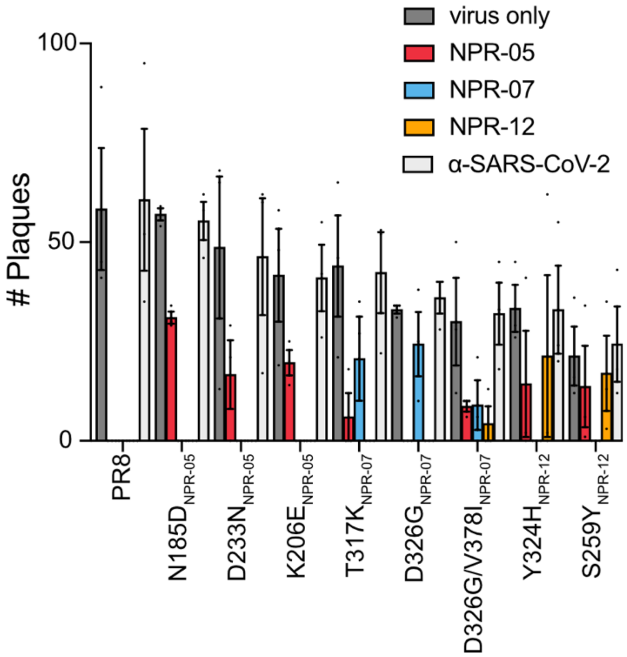

Supplementary Figure 4. Quantification of plaques in plaque reduction assay in Fig. 3C. Data are shown as mean ± SEM, (N = 3).
